# Genome-wide identification and evolution of ATP-binding cassette transporters in the ciliate *Tetrahymena thermophila*: A case of functional divergence in a multigene family

**DOI:** 10.1186/1471-2148-10-330

**Published:** 2010-10-27

**Authors:** Jie Xiong, Lifang Feng, Dongxia Yuan, Chengjie Fu, Wei Miao

**Affiliations:** 1 Key Laboratory of Aquatic Biodiversity and Conservation, Institute of Hydrobiology, Chinese Academy of Sciences, Wuhan, 430072, PR China; 2 State Key Laboratory of Freshwater Ecology and Biotechnology, Institute of Hydrobiology, Chinese Academy of Sciences, Wuhan, 430072, PR China; 3 Program in Systematic Biology, Evolutionary Biology Centre, Uppsala 75236, Sweden; 4 College of Food Science and Biotechnology, Zhejiang Gongshang University, 149 Jiaogong Road, Hangzhou 310035, PR China; 5 Graduate School of Chinese Academy of Sciences, Beijing, 100049, PR China

## Abstract

**Background:**

In eukaryotes, ABC transporters that utilize the energy of ATP hydrolysis to expel cellular substrates into the environment are responsible for most of the efflux from cells. Many members of the superfamily of ABC transporters have been linked with resistance to multiple drugs or toxins. Owing to their medical and toxicological importance, members of the ABC superfamily have been studied in several model organisms and warrant examination in newly sequenced genomes.

**Results:**

A total of 165 ABC transporter genes, constituting a highly expanded superfamily relative to its size in other eukaryotes, were identified in the macronuclear genome of the ciliate *Tetrahymena thermophila*. Based on ortholog comparisons, phylogenetic topologies and intron characterizations, each highly expanded ABC transporter family of *T*. *thermophila *was classified into several distinct groups, and hypotheses about their evolutionary relationships are presented. A comprehensive microarray analysis revealed divergent expression patterns among the members of the ABC transporter superfamily during different states of physiology and development. Many of the relatively recently formed duplicate pairs within individual ABC transporter families exhibit significantly different expression patterns. Further analysis showed that multiple mechanisms have led to functional divergence that is responsible for the preservation of duplicated genes.

**Conclusion:**

Gene duplications have resulted in an extensive expansion of the superfamily of ABC transporters in the *Tetrahymena *genome, making it the largest example of its kind reported in any organism to date. Multiple independent duplications and subsequent divergence contributed to the formation of different families of ABC transporter genes. Many of the members within a gene family exhibit different expression patterns. The combination of gene duplication followed by both sequence divergence and acquisition of new patterns of expression likely plays a role in the adaptation of *Tetrahymen *a to its environment.

## Background

The ATP-binding cassette (ABC) transporter superfamily of genes is one of the largest in the genomes of both bacteria and eukaryotes [1]. Using hydrolysis of ATP to ADP to generate energy, ABC transporters move a wide variety of substrates across membranes, including ions, sugars, amino acids, polypeptides, toxic metabolites, xenobiotics, and drugs. Therefore, they provide nutrients to a cell as well as protect it from both internally produced and exogenous toxins [2,3]. Eukaryotic ABC transporters usually consist of two types of domains, a transmembrane domain (TMD) and a nucleotide-binding domain (NBD). Many ABC proteins include two of each type of domain and are so-called full transporters. Others are half transporters, containing one TMD and one NBD, and generate a functional unit by forming a homo- or heterodimer [2]. Based on their primary sequences and the organization of their domains, the human ABC genes were classified into seven families, from ABCA to ABCG [4]; an eighth ABCH family was discovered in the analysis of the *Drosophila melanogaster *genome [5].

In humans, mutations of many ABC genes are linked to hereditary disorders, such as adrenoleukodystrophy and cystic fibrosis [5,6], and proteins coded for by many genes in the ABC families B, C and G function as drug efflux transporters [7]. In parasites, products of ABC genes have been implicated as factors contributing to resistance against chemotherapeutics [8], and in insects, ABC genes have been linked to pesticide resistance [9-11]. These medical and toxicological roles make ABC transporters important in pharmacological research [12], therapeutic applications [13], and toxicology [14]. Extensive investigations of ABC transporters in bacteria and multicellular eukaryotes have been done [5,15-22], but studies in unicellular eukaryotes other than yeasts have been limited to parasitic species [23,24]. Clearly, the ABC transporter superfamily in unicellular free-living species requires additional study.

*Tetrahymena *is a free-living ciliated protist found in freshwater environments around the world [25]. At the cellular level, its structural and functional complexity is equal to or greater than that of individual metazoan cells. Studies on *Tetrahymena *have led to numerous scientific breakthroughs, and a number of molecular genetic technologies and genomic resources have recently been developed [26-28]. In particular, the *Tetrahymena *Genome Database (TGD, http://www.ciliate.org) and *Tetrahymena *genome expression database (TGED, http://tged.ihb.ac.cn) [28,29] provide the opportunity for analysis of both the functional and evolutionary characteristics of gene families at the genomic level in this model organism [30].

In the present study, we identified 165 ABC transporter genes in the *Tetrahymena thermophila *macronuclear genome. The locations of introns, evolutionary relationships and expression patterns of these genes were characterized. A detailed analysis showed that the evolutionary and functional divergence of *Tetrahymena *ABC transporters has resulted from a combination of gene duplication, mutation, pseudogenization, and changes in gene expression.

## Results and Discussion

### Identification and classification of ABC transporter genes in *T. thermophila*

**Figure 1:**
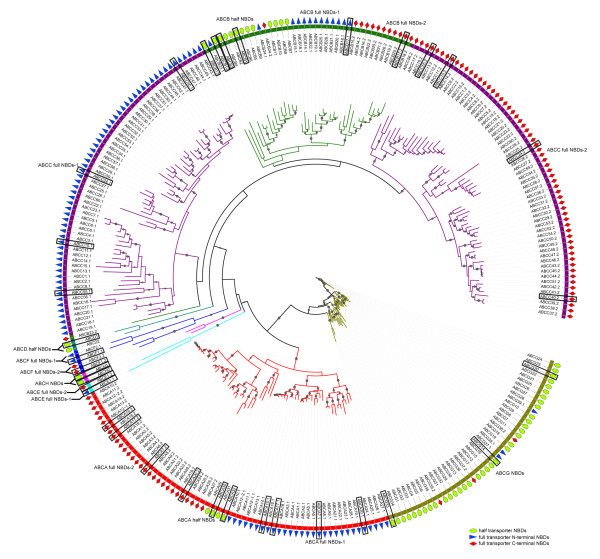
**Unrooted ML tree of ABC transporter NBDs in *T. thermophila***. Each color represents a family of ABC transporters. Genes that have an ortholog in the genome of *Paramecium *are marked with black panes. Bootstrap values > 50% consensus are marked with gray circles, and their sizes indicate the levels of consensus.

A total of 165 putative ABC transporter genes were identified in *T. thermophila *(Additional file 1), making it the largest superfamily of ABC transporter genes described in any species to date. All of the genes could be grouped into eight families based on organization of domains, BlastP scores, and placement in phylogenetic trees. Their phylogenetic relationships are shown in the ML tree (Figure 1).

**The ABCA family **comprises 29 full transporters and 4 half transporters. The gene model for one full ABC transporter gene, *ABCA12 *(TGD accession NO: TTHERM_00694300), was predicted incorrectly by TIGR. Our RT-PCR results showed that this "monster" gene was, in fact, two full-transporter genes, i.e., *ABCA12-1 *and *ABCA12-2 *(data not shown). In vertebrates, the ABCA family is composed entirely of full transporters, and in the plant *Arabidopsis *all are half transporters [31]. However, both full and half transporters have been found in another protist, *Dictyostelium *[32]. No *ABCA *genes have been identified in yeast [33] (Table 1). Three ABCA genes (*ABCA1*, *ABCA8*, and *ABCA15*) in *Tetrahymena *are highly similar to the *suABCA *gene in sea urchins, which was found in sperm membrane vesicles and is involved in changing the immature sperm membrane to produce the terminally differentiated mature spermatozoa [34]. These three *Tetrahymena *ABCA genes were markedly up-regulated during starvation, and the high levels of expression were maintained during conjugation. Starvation induces physiological changes that prepare cells of different mating-types to form mating pairs [35]; therefore, these ABCA genes may be involved in inducing and/or sustaining conjugation.

**Table 1:** Comparison of ABC transporters in *Tetrahymena thermophila *and other eukaryotes

Family	Structure	Tt	Pt	Bb	Py	Dd	At	Sc	Hs
ABCA	(TMD-NBD)_2_	29^a^	20	0	0	7	0	0	13
	TMD-NBD	4	4	0	0	5	17^b^	0	0
ABCB	(TMD-NBD)_2_	13	7	0	1	2	22	1	4
	TMD-NBD	13	7	4	6	7	5	3	7
ABCC	(TMD-NBD)_2_	60	46^c^	4	1	14	15	7	12
ABCD	(TMD-NBD)_2_	0	0	0	0	0	1	0	0
	TMD-NBD	2	4	0	0	3	1	2	4
ABCE	NBD-NBD	1	2	1	1	1	2	1	1
ABCF	NBD-NBD	2	2	1	1	4	5	5	3
ABCG	(TMD-NBD)_2_	3	0	0	0	15	13	10	0
	TMD-NBD	36	17	0	1	6	29	1^d^	5
Other^e^	NBD	2	3	0	3	4	15	2	2
Total	-	165	112	10	14	68	125	32	50
Number of Genes	-	24725	39642	3671	5878	12500	25498	5538	22287
Percent	-	0.667%	0.283%	0.272%	0.238%	0.544%	0.490%	0.577%	0.224%

Tt = *Tetrahymena thermophila*, Pt = *Paramecium tetraurelia*, Bb = *Babesia bovis *(has the minimum predicted gene number in sequenced apicomplexans), Py = *Plasmodium yoelii yoelii *(has the maximum predicted gene number in sequenced apicomplexans), Dd = *Dictyostelium discoideum*, At = *Arabidopsis thaliana*, Sc = *Saccharomyces cerevisiae*, Hm = *Homo sapiens*

^a ^one incorrectly predicted gene was identified

^b ^includes a gene annotated as (TMD-NBD)_2 _in a previous report, but revised as TMD-NBD in the current version of the annotation

^c ^includes 5 genes with a structure TMD-NBD and 2 genes with a structure NBD

^d ^this gene has three TMDs and one NBD

^e ^includes the ABCH family of gene

**The ABCB family **consists of 13 full transporters and 13 half transporters. Among those, one full transporter, *ABCB23 *(TGD accession NO: TTHERM_00803570), possesses both one ABCB-like NBD (E-value: 6e^-61^) and one ABCC-like NBD (E-value: 3e^-50^). The mixed structure of this gene was verified by sequencing its cDNA between the first NBD and second TMD (Additional file 2). The *ABCB23 *gene was grouped into the ABCB family according to the higher similarity of its NBD (lower E-value) to ABCB-like NBD.

Two ABCB genes (*ABCB12 *and *ABCB13*) in *Tetrahymena *were predicted to have a mitochondrial localization by the Predotar program (http://urgi.versailles.inra.fr/predotar/predotar.html). The half transporter ABCB12 has an ABC_ATM1 domain (cd03253, E-value: 2e^-65^) that resembles the NBD of human ABCB7 and the yeast ATM1 gene, both of which are involved in the transport of the Fe/S binding protein into mitochondria [36]. There is an ABC_MTABC3 domain (cd03249, E-value: 1e^-63^) in ABCB13 that resembles the NBD of human ABCB6, which has been shown to be associated with iron transport and is located in the mitochondrial membrane [37].

The human *ABCB1 *gene MDR1 (multidrug resistance gene) encodes a well-studied p-glycoprotein that has been shown to confer resistance to or transport a wide variety of anticancer agents. The MDR protein is thought to transport both endo- and xenobiotics and protect cells from toxic agents [38]. The *ABCB15 *(TGD accession NO: TTHERM_00240450) of *T. thermophila *shares a 32.3% amino acid identity with MDR1, which is higher than that between the human p-glycoprotein and its ortholog *STE6 *in the yeast (26.2%) [39]. A protein composed of both 66 and 96 kDa subunits has been reported to function in *T. pyriformis *as an efflux pump similar to mammalian MDR1 [40,41], and a putative p-glycoprotein in *T. pyriformis *(GenBank accession No. CAD55936) has been described. The *ABCB15 *of *T. thermophila *shares a 35.2% amino acid identity with a putative p-glycoprotein of *T. pyriformis *and is induced by DDT, but not by either TCDD or β-estradiol (Additional file 3). Thus, the *ABCB15 *protein might function as a substrate-specific MDR,

**The ABCC family **comprises 60 full transporters and thus represents the largest family of ABC transporters in *T. thermophila*. In the human genome, nine *ABCC *genes encode the multidrug resistance proteins (MRPs), which contain a core domain of TMD similar to p-glycoprotein, with the difference that some of them have an extra TMD at their N-terminal region to form the structure TMD-TMD-NBD-TMD-NBD. MRPs transport xenobiotics coupling glutathione (GSH), which is a major feature distinguishing them from p-gp; therefore MRPs also are recognized as GS-X pumps [38,42]. In *T. thermophila*, no extra TMD was found at the N-terminal region of *ABCC *proteins, but an extra GST_Mu domain was present at the C-terminal region of *ABCC52 *(Additional file 1). Xenobiotics are fused with GSH by glutathione *S*-transferases at phase II, and then the complexed substances are transported by MRPs at phase III, suggesting a coordinated action of GSTs and MRPs [43]. *ABCC52 *is a MRP-like gene and the GST_Mu domain may indicate that it functions similarly to recognize such GST-complexed substances.

**The ABCD family **contains only 2 half transporters. The ABCD families of most other organisms also consist of a low number of half transporters except for that of the plant *Arabidopsis thaliana*, which includes both a full and a half transporter [44] (Table 1). Members in this family that have been studied are all targeted to the peroxisome, where they regulate the transport of long-chain fatty acids [45]. In yeast, two ABCD half transporters (Pxa1p and Pxa2p) were found, and these two half transporters form a heterodimer that functions in the β-oxidation of long-chain fatty acids [46]; In humans, there are four ABCD genes [47], the human ABCD1 is responsible for the X-linked form of adrenoleukodystrophy as a homodimer [48]. The *ABCD1 *and *ABCD2 *genes of *T. thermophila *exhibited the same pattern of expression (Pearson Correlation Coefficient, PCC = 0.948) during the physiological/developmental stages of growth, starvation and conjugation (Additional file 4), suggesting that a heterodimer may also be their functional form.

**The ABCE and ABCF families **each contain only two NBDs without TMDs in *T. thermophila*. Almost all eukaryotes also have only one *ABCE *gene except *A. thaliana*, which has two [31]. In *Tetrahymena*, also one ABCE gene has been identified. In animals, ABCE has been shown to inhibit RNase L, a double-stranded RNA nuclease, and is referred to as RLi [49]. Many intron positions within *ABCE1 *genes are conserved in animals and plants, especially in vertebrates (Additional file 5); therefore, it has been suggested that most of these introns (e.g., those in human, mouse and rat ABCE1) were ancestral in nature and that many introns have been lost in other species (e.g., *Ciona intestinalis*, *Anopheles gambiae*, *Drosophila melanogaster*, *Caenorhabditis elegans*, *Dictyostelium discoideum*, *Plasmodium falciparum*) [15]. The ABCE1 of *T. thermophila *contains 6 introns; however, it shares no intron positions with ABCE1s of animals and only one with that of *Arabidopsis*, suggesting that the timing and mechanism of intron gain and loss in the *ABCE1 *gene of *T. thermophila *may have differed from those of opisthokonts and plants. For the ABCF family, a well studied gene is the GCN20 of yeast, which has been shown to regulate translation in amino acid-starved cells by interaction with eukaryotic initiation factor 2 (eIF2) and ribosomes [50]. In *Tetrahymena*, two ABCF genes were found; one of the two genes (TGD accession NO: TTHERM_01014620) showed a high expression level in the three stages of *T. thermophila *life cycle, indicated a constitutive function of this gene.

**The ABCG family **includes 3 full transporters and 36 half transporters. Members of the ABCG family are structured as NBD-TMD or NBD-TMD-NBD-TMD that have a TMD at the C-terminal region of NBD, an orientation inverse to that of the ABCA, ABCB, ABCC and ABCD families. Human *ABCG2*, first identified in cells from placentas and breast cancers, is a multidrug resistance ABC transporter gene different from both MDR1 or MRP1 and is known as breast cancer resistant protein (BCRP). A half transporter, BCRP functions as a homodimer or tetramer bridged with disulfide bonds [51,52]. Expression of *ABCG25 *is induced by DDT *T. thermophila*, and it shares 31.8% amino acid identity with BCRP, suggesting that it might have the same basic function (Additional file 3).

**The ABCH family **was identified in *Drosophila *[47], and lacks members in mammals and *C. elegans*. The function of ABCH is unknown. *Tetrahymena *has 2 ABCH genes, both of which have a single NBD. The *ABCH1 *gene of the social amoeba *D. discoideum *has the same structure [32]. However, genes of the ABCH family encode a half transporter with an NBD-TMD structure in *D. melanogaster *and *A. gambiae *[47].

### The evolution of ABC transporters in *Tetrahymena*

All N-terminal NBDs (NBDs-1) and C-terminal NBDs (NBDs-2) of full transporters and NBDs of half transporters (NBDs-half) were used to construct the ML tree (Figure 1). It showed that NBDs in the same family clustered together except for the ABCC family (Figure 1). The relationship of the eight families was similar to that previously reported in *Dictyostelium *[32]. Comparative genomics analyses of ABC transporters suggested that the ancestral ABC transporters arose before the differentiation of prokaryotes and eukaryotes [53-55]. In another analysis using 19 evolutionarily diverse eukaryotes, those NBDs in the same family still cluster together (unpublished result). These results suggest that the eight ABC transporter families may have diverged prior to the last universal common ancestor (LUCA) of existing eukaryotes.

**Figure 2:**
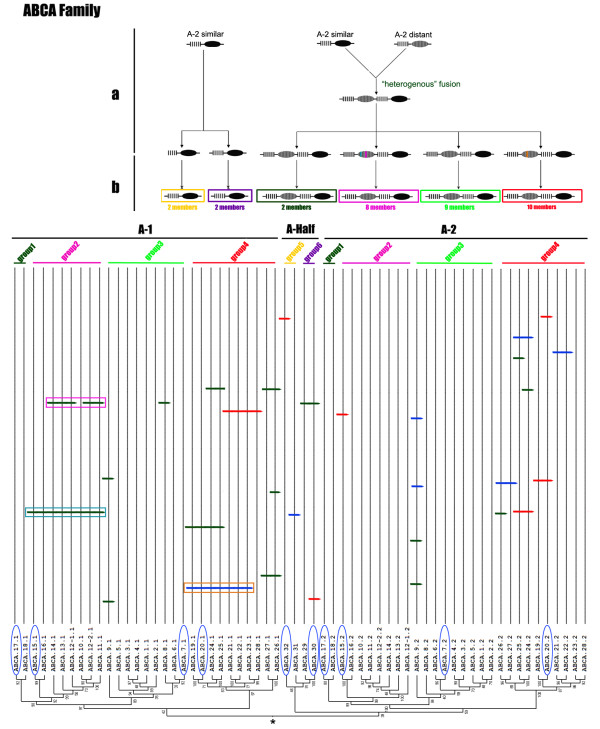
**Phylogenetic tree and a proposed evolutionary scheme of the ABCA family**. Above: the proposed events in the evolution of the ABCA family. a, putative full transporter formation process and divergence of the ABCA transporter with gain and loss of introns, the colored vertical bars in the NBD region represent conserved introns; b, divergence of the ABCA transporter gene to give rise to each group. Below: part of tree from Figure 1 with introns locations of NBDs, the asterisk represent the root in the midpoint. Each group was marked as a line with the same color to the pane in b; genes that have a *Paramecium *ortholog were marked with a blue ellipse.

**Figure 3:**
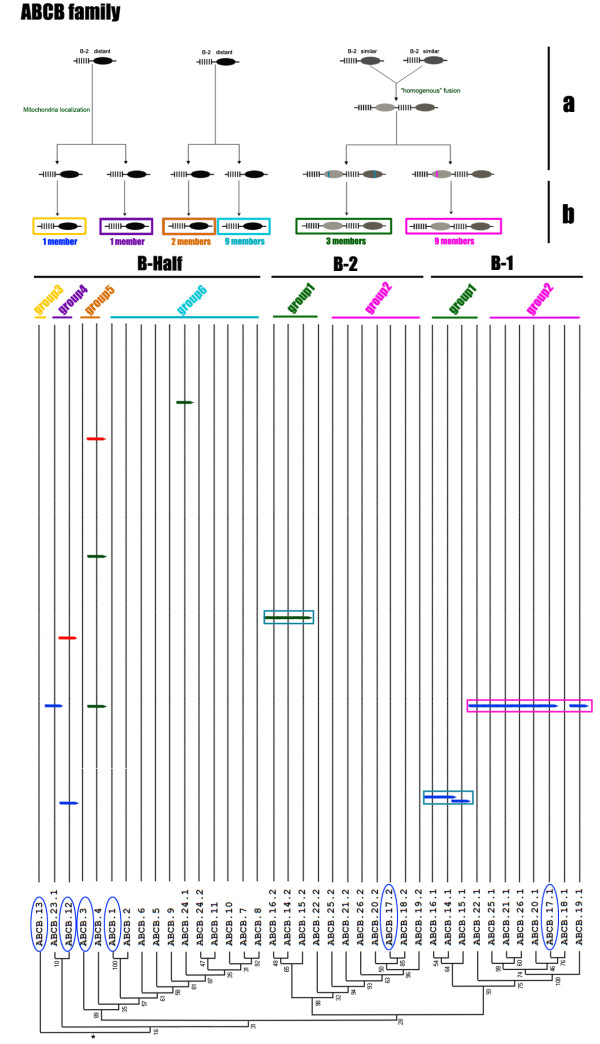
**Phylogenetic tree and a proposed evolutionary scheme of ABCB family**. Above: the proposed events in the evolution of the ABCB family. a, putative full transporter formation process and divergence of the ABCB transporter with gain and loss of introns, the colored vertical bars in the NBD region represent conserved introns; b, divergence of the ABCB transporter gene to give rise to each group. Below: part of tree from Figure 1 with introns locations of NBDs, the asterisk represents the root in the midpoint. Each group was marked as a line with the same color to the pane in b; genes that have a *Paramecium *ortholog were marked with a blue ellipse.

**Figure 4:**
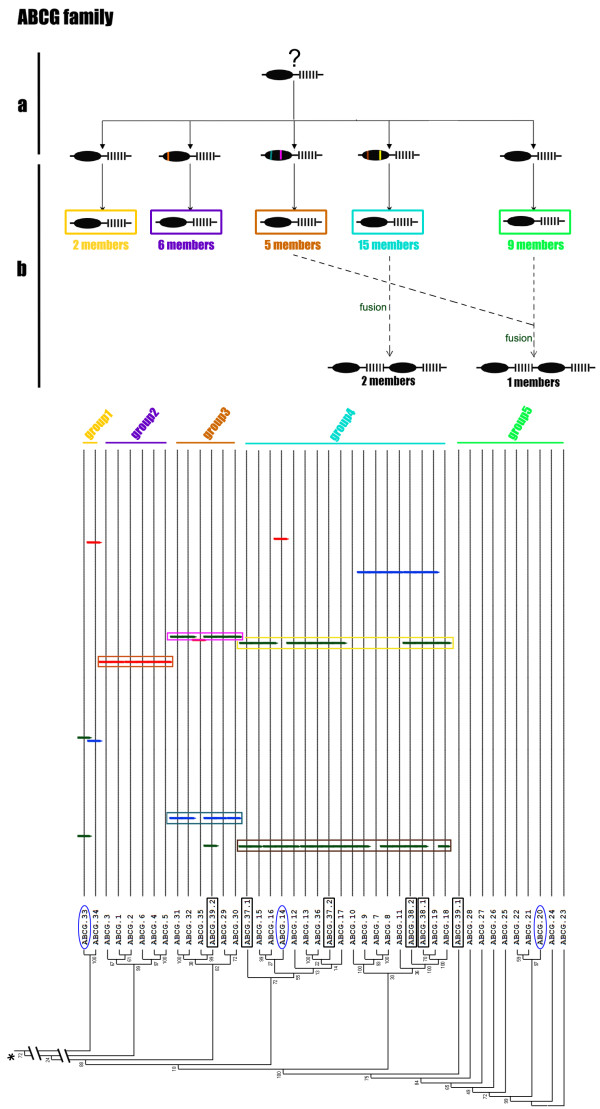
**Phylogenetic tree and a proposed evolutionary scheme of ABCG family**. Above: the proposed events in the evolution of the ABCG family. a, divergence of the half transporter with intron gain and loss, and the colored vertical bars in the NBD region represent conserved introns; b, divergence of the ABCG half transporter gene to give rise to each group, and possible formation process of ABCG full transporters (dash line). Below: part of tree from Figure 1 with introns locations of NBDs, the asterisk represent the root in the midpoint. Each group was marked as a line with the same color to the pane in b. The full transporter NBDs of ABCG are marked with a black pane in the phylogenetic tree; genes that have a *Paramecium *ortholog were marked with a blue ellipse.

Many proteins are composed of at least two domains, and it has become clear that the formation of new domain combinations is an important mechanism in protein evolution [56]. In most cases, but not always, the eukaryotic ABC transporters are composed of two or four domains through various combinations of TMDs and NBDs. Half transporters are composed of a single TMD domain fused to an NBD domain, a structural organization that could be symbolized as TMD-ABC or ABC-TMD (ABCG family) depending on the N- or C-terminal location of the TMD domain. Full-size transporters are probably generated by duplication and fusion of "half-size" transporters and are symbolized as [TMD-NBD]_2 _and [NBD-TMD]_2 _(ABCG family) [55,57-59]. In our analysis, three phylogenetic relationships were found among the three kinds NBDs (NBDs-1 and NBDs-2 from full transporters and NBD-Half from half transporters) in an unrooted tree, corresponding to the ABCA, ABCB and ACBG families respectively. In the ABCA family, NBDs-2 (A-2) showed a distant relationship to NBDs-1 (A-1) and closer relationship to NBDs-Half (A-Half) (Figure 2), which indicated the possibility that the ABCA full transporter fused from two distinct half transporters, which we refer to as "heterogenous" fusion. In the ABCB family, NBDs-2 (B-2) showed a close relationship to NBDs-1 (B-1) and a more distant relationship to NBDs-Half (B-Half) (Figure 3), which indicated the possibility that the ABCB full transporter formed by fusion of two high similarity half transporters, referred to as "homogenous" fusion. The ABCG family possesses three full transporters with a reverse domain organization. Neither NBDs-1 (G-1) nor NBDs-2 (G-2) associated into separate cluster but, instead, were dispersed among the cluster of NBDs-Half (G-Half) (Figure 4). Since there are no ABCG full genes in the *P. tetraurelia *genome, this result indicates that the *T. thermophila *ABCG full transporters originated after these two species diverged. NBDs-1 and NBDs-2 of two full genes (ABCG37 and ABCG38) are in the same group (group4) (Figure 4), while two NBDs of the other ABCG full transporter (ABCG39) cluster into two groups (group3 and group5). Although the ancestor could not be determined in an unrooted tree, these three pairs of NBDs seem to correspond to "homogenous" and "heterogenous" fusions. In addition, the ABCC family members were all full transporters with distant relationship of NBDs-1 and NBDs-2, also may have undergone a heterogenous fusion process.

**Figure 5:**
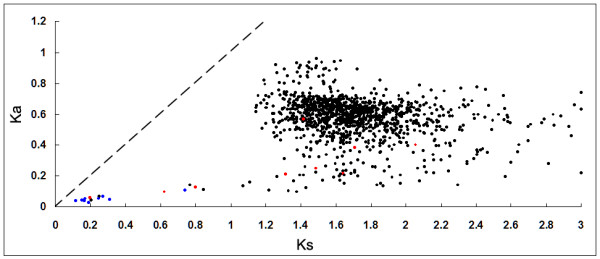
**Scatter plot of Ka versus Ks**. Red dots, pairs of two tandem duplicates; blue dots, pairs of multiple tandem duplicates with a low value of Ks; the dashed line represents Ka = Ks (neutral selection); several pairs with Ks > 3 were omitted.

Possession of a large number and variety of ABC transporters is a significant characteristic of *T. thermophila*, and we carried out an analysis to discover whether this results from numerous, lineage-specific duplications and when these duplications occurred. First, a phylogeny of NBDs was used to investigate the evolution of all of the 165 *T. thermophila *ABC transporter genes, using both full transporters (NBDs-1 and NBDs-2) and half transporters (NBDs-half) (Figure 1). A strong correlation was observed between the conservation of intron positions and phylogenetic relationships of ABC transporters, except for some scattered examples which may have resulted from recent intron-gain events. Next, full and half transporters in the ABCA, ABCB, ABCC, and ABCG families were divided into groups. Within each group, the locations of introns were conserved, indicating that intron gain and loss had occurred before the evolutionary expansion of each family. The ABCA, ABCB, ABCC, and ABCG families were massively expanded, containing 17 clusters of tandem repeat genes that comprised a total of 58 genes (Table 2). The ABCG family had the most clusters, six with a total of 29 genes, and its largest single cluster had 11 tandem genes. The sequence identities of proteins in each tandem cluster ranged widely (50%-80%) and correlated with values of Ks for these tandem genes. However, several tandem repeats had a low Ks (0.1169-0.3109) (Figure 5), and notably, genes within tandem clusters were more closely related to each other than to those in other clusters. This suggests either that duplication events had occurred relatively recently within the clusters of tandem genes or that some mechanism results in the concerted evolution of these genes.

**Table 2:** Numbers of clusters with tandem repeats in four large families of ABC transporter gene in *Tetrahymena*

Family	2 repeats	3 repeats	4 repeats	5 repeats	6 repeats	11 repeats	n
ABCA	2	-	-	-	1	-	3
ABCB	3	-	-	-	-	-	3
ABCC	3	1	1	-	-	-	5
ABCG	2	1	-	1	1	1	6

The ABCA family can be divided into 6 groups including two groups of half transporters (groups 5-6 in Figure 2) and four groups of full transporters (groups1-4 in Figure 2), suggesting that *Tetrahymena *inherited at least 6 ABCA transporter genes from its ancestor. Regarding the expansion of full transporter genes, it was significant that the tree topologies of full transporter NBDs-1 and NBDs-2 were surprisingly similar (Figure 2). This suggests that these genes evolved as full transporters first and then became expanded. Thus, three genes ancestral to full transporters expanded to form groups1-4, containing 2, 8, 9 and 10 genes, respectively (Figure 2b).

Similar phenomena also were observed in the ABCB (Figure 3) and ABCC families (data not shown). The ABCG family differed from the others in being composed mainly of expanded half transporters--5 ancestral genes expanded to 2, 6, 5, 15 and 9 genes, respectively (Figure 4b). More than 90% of the ABCG genes in *Tetrahymena *are half transporters that are more similar to one another in their sequences than to other ABC transporter families (data not shown), which indicates either very recent expansion, higher negative selection or highly concerted evolution of these genes.

After the phylogenetic analysis of NBDs within *Tetrahymena*, we compared the detailed compositions of ABC transporter families in *Tetrahymena *to those of other eukaryotes (Table 1). *Tetrahymena*, with a predicted proteome of 24725 genes, possesses 53 more ABC transporter genes than those of *P. tetraurelia*, its closest sequenced free-living ciliate which has been shown to have undergone three whole genome duplications (WGDs) [60] resulting in a predicted proteome of 39642 genes. *Tetrahymena *and *Paramecium *probably branched off ~800 Mya, yet only 23 orthologs of ABC transporters between *Paramecium *and *Tetrahymena *were found using a reciprocal Blast search (see methods); therefore, 86% of ABC genes in *Tetrahymena *appear to have evolved after divergence of these two species. The pairwise Ks values of 7 sets of ABC transporter genes (see methods) fell mainly between 1.2-2.2 (Figure 5). Rebecca et al (2006) compared the evolutionary rates of proteins among animals, plants and ciliates using Ka/Ks and concluded that the genomic architecture of ciliates has experienced the highest rate of evolution, at least 1.6-2.6 times greater than in any other major group of eukaryotes investigated to date [61]. An overall rate of 9.76 mutations per silent site per billion years in *Tetrahymena *can be calculated using the overall rate of mutations in *Arabidopsis *(6.1 per silent site per billion years) as a basis for calculation, with a normalization of 1.6 fold, [62]. If this is correct, then most of the duplications of ABC genes in *T. thermophila *probably occurred between 61.5-112.7 Mya ago. However, it should be noted that this is a preliminary estimate, and further analysis of the general rate of protein evolution at the genomic level will be required for a more precise calculation.

Comparisons of *Tetrahymena *with other alvelolate protists, such as the apicomplexans *Babesia bovis *and *Plasmodium yoelii*, or more distantly related model organisms, such as *S. cerevisiae, D. discoideum*, *A. thaliana *and *H. sapiens*, also suggest that the superfamily of ABC transporters in *Tetrahymena *not only has a higher absolute number of genes but also represents a higher percentage of the predicted proteome (Table 1). The large size of this family in *Tetrahymena *may result from either an increased rate of gene duplication or a low rate of loss. Moreover, it has been shown in *Tetrahymena *that only genes associated with sensing of and responding to environmental changes (e.g., kinases, voltage-gated ion channels, P-type ATPases and transporter genes) have undergone such large-scale, lineage-specific expansions resulting in a higher number of genes than in other eukaryotic taxa, including the other free-living protists [27]. This suggests that the enlargement of several ABC transporter families in *T. thermophila *resulted from relatively a high duplication rate.

### Functional divergence of ABC gene duplicates in *Tetrahymena*

Gene duplication is a primary source of new genes with novel or altered functions [63], and a comprehensive classification of all models for the evolution and maintenance of gene duplication has been provided in a recent review [64]. A study of the patterns of expression is one important measure of functions of a gene that could facilitate understanding of the genetic basis of evolutionary change [65,66]. The extensive duplications of genes in the ABC transporter superfamily of *Tetrahymena *provide a particularly good opportunity to investigate the functional consequences of gene duplication in a single organism.

**Figure 6:**
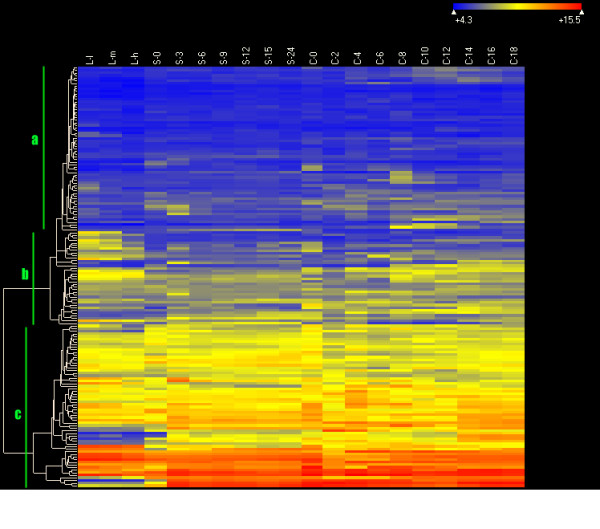
**Heat map of the expression of ABC transporter genes in *Tetrahymena***. The levels of expression are illustrated by different grades of color as determined from microarray data indicated along the top (From left to right). The color scale is as follows: dark color, low expression; light color, high expression. Levels of expression were obtained for 20 points in time during three physiological/development stages of the life cycle of *Tetrahymena*: For growing cells, **L-l**, **L-m **and **L-h **correspond to ~1×10^5 ^cells/ml, ~3.5×10^5 ^cells/ml and ~1×10^6 ^cells/ml, respectively. For measurements of expression during starvation, ~2×10^5 ^cells/ml were collected at intervals of 0, 3, 6, 9, 12, 15 and 24 hours (referred to as **S-0, S-3, S-6, S-9, S-12, S-15 **and **S-24, **respectively). For measurements of expression during conjugation, equal volumes of B2086 and CU428 cells were mixed together in culture, and samples were collected at intervals of 0, 2, 4, 6, 8, 10, 12, 14, 16 and 18 hours after mixing (referred to as **C-0, C-2, C-4, C-6, C-8, C-10, C-12, C-14, C-16 **and **C-18, **respectively). The following three categories could be identified: a, silent genes or those with low expression levels (some points may silent); b, genes highly expressed only for specific conditions; c, genes with continuous high expression levels.

Figure 6 shows a heat map illustrating the overall expression profiles of ABC transporters in *T. thermophila *during different physiological/developmental conditions. Three categories could be identified based on cluster analysis: 1) silent genes or those with low expression levels (some points may silent) (Figure 6a); 2) genes expressed only under specific conditions (Figure 6b); 3) genes with high expression levels for all the experimental conditions (Figure 6c). Seven sets of duplicates of ABC genes in the four largest families (ABCA, ABCB, ABCC and ABCG) were selected for analysis of the evolution of their gene-expression patterns. Among these, one set was fit best with the Non-phylogenetic/Free model, and the others all fit best with the Pure-phylogenetic/free model (Table 3). The Pure-phylogenetic model assumes that changes in gene expression occurred on every branch of the phylogeny and that there were neither loss of functions within a gene family in the course of this process nor sporadic changes in gene expression [66]. Thus, either pseudogenization or functional divergence might have occurred in the cases of those six sets of ABC transporter genes.

**Table 3:** The best model of evolution of gene expression for seven sets of genes

Sets of genes	models	AIC value
ABCA-Set1	Pure-Phylogenetic/Free	938.55
ABCA-Set2	Non-Phylogenetic/Free	394.963
ABCB-Set1	Pure-Phylogenetic/Free	317.005
ABCB-Set2	Pure-Phylogenetic/Free	408.603
ABCC-Set1	Pure-Phylogenetic/Free	586.047
ABCC-Set2	Pure-Phylogenetic/Free	1214.151
ABCG-set1	Pure-Phylogenetic/Free	1131.114

**Figure 7:**
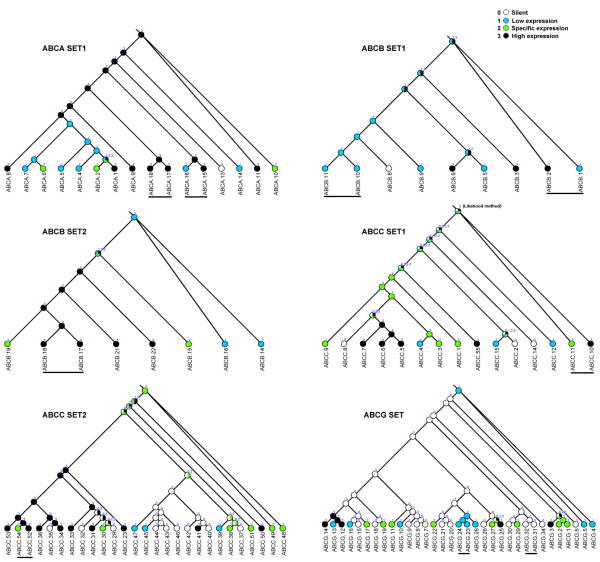
**A phylogeny of six sets of ABC gene duplicates in *T. thermophila *with inferred states of ancestral expression**. Tandem duplicates are underlined in black. The following numbers represent four different states of expression: 0 = Silent, 1 = Low expression (some points may silent), 2 = Specific high expression and 3 = Continuous high expression.

We attempted to separate the effects of different modes of duplicate retention by categorizing the expression profile into four types (see methods) for the seven sets of gene duplicates and inferring their ancestral expression states for a comparison. Tandem duplicates of ABC transporters were chosen to represent relatively recent duplications, there being nine such duplicates distributed in six sets of genes (Figure 7). In addition, analyses of those gene sets were performed where applicable, including calculation of Ka/Ks values and sites under positive selection, the possibility of forming a heterodimer, and the inducibility to toxic exposure as accessory evidence. The following four different models could be inferred from these nine pairs of duplicates (Table 4).

**Table 4:** The best fitted model of the nine tandem duplicates

Gene pair	ABCA				ABCB						ABCC				ABCG			
	
	ABCA15	ABCA16	ABCA17	ABCA18	ABCB1	ABCB2	ABCB10	ABCB11	ABCB17	ABCB18	ABCC10	ABCC11	ABCC52	ABCC54	ABCG23	ABCG24	ABCG31	ABCG32
**Taxon state**	3	1	3	3	1	3	1	1	3	3	3	2	3	2	1	1	0	0
**Ancestral state**	3		3		1 or 3		1		3		2		3		1		0	
**Ks**	0.62		1.71		0.80		1.41		2.05		1.31		1.64		0.20		1.49	
**Ka/Ks**	0.16		0.22		0.16		0.39		0.20		0.16		0.13		0.29		0.17	
**Possible heterodimer**	N		N		N		Y		N		N		N		Y		N	
**Induced by toxin**	N	N	N	N	N	N	N	N	Y (DDT)	N	N	N	N	N	N	N	N	N
**Positive selection sites**	31		26		0		0		2		9		2		4		0	
**Best model**	neofunctionalization		neofunctionalization		subfunctionalization		dosage balance		neofunctionalization		neofunctionalization		uncertain model		dosage balance		pseudogenization	

Taxon or ancestral expression state: 0 = Silent, 1 = Low expression (some points may silent), 2 = Specific high expression and 3 = Continuous high expression.

Y = yes, N = no.

• Neofunctionalization for four pairs. 1) The ACBA15-ABCA16 pair is likely to be undergoing a coding level neofunctionalization, since many sites were detected under positive selection for this pair (Table 4). The loss of expression in one copy could have been shaped by a reduction of the formerly high constitutive expression, to perform a novel function related to, for example, a rare or low-concentration xenobiotics. 2) the ABCA17-ABCA18 pair showed the same expression pattern for the ancestral state and two descendants. Similar to the ABCA15-ABCA16, this pair has undergone coding-level neofunctionalization given the relatively high age of the duplication and a large number of sites under positive selection (Table 4). 3) the ABCB17-ABCB18 pair had the same type of expression in the ancestral state as the two genes in the pair (Table 4), whereas the ABCB17 gene could be induced by exposure to DDT (unpublished data), suggesting that ABCB17 might have evolved a new functional role. 4) the ABCC10-ABCC11 pair was inferred as having an ancestral state of high expression for specific conditions still found in the ABCC11 gene, but ABCC10 showed a continuous high level of expression (Table 4). Furthermore, positive selection on some sites also was detected in the four pairs (Table 4); therefore, the neofunctionalization model was determined to be the best one.

• Subfunctionalization for one pair. The subfunctionalization model implies that duplicates partitioned the function of the ancestral gene and underwent a reciprocal rapid, partial loss of function [67,68]. Two expression patterns would be consistent with this model: ancestral ubiquitous expression followed by both duplicates specializing their expression patterns, or high ancestral expression levels with low duplicated pair expression levels (that add up to the previous high level). In our analysis, the ABCB1-ABCB2 showed a relatively recent duplication (with low Ks), and more important, the ancestral state may have shown both expression patterns of the descendants prior to duplication (Table 4), therefore, this pair is the most probable duplicates for subfunctionalization. However, simple gradual pseudogenization is another possible explanation for this pair.

• Dosage balance for two pairs. The dosage balance model implies a positive selection for fixation of the duplicates, with both descendants maintaining the original function showing signs of negative selection at the coding level in preservation phase. This model may apply to genes involved in transport [69] and protein-protein interactions [70]. With low Ka/Ks values, two pairs (ABCB10-ABCB11 and ABCG23-ABCG24) showed the same type of expression in the ancestral state as in both duplicates, and these two pairs possibly form heterodimers based on their co-expression PCC values (Table 4), indicating a dosage balance model for these two pairs. The expressed macronuclear genome of *Tetrahymena *is effectively polyploid (45 copies of each gene on average); however, the retention of both members of a gene pair after duplication to enable an increase in the level of response to match exposure to substances (i.e., increased dosage) may still play a role in the evolution of ABC transporter genes in *T. thermophila.*

• Pseudogenization for one pair. The tandem duplicate ABCG31-ABCG32 showed a silent state during the entire life cycle of the *T. thermophila *(Table 4), and no evidence of transcriptional inducibility from exposure to several toxic substances. The parsimonious ancestral expression state of this pair was also silent (Figure 7). Since the duplication is fairly old and the pairwise Ka/Ks hints at negative selective pressure operating until quite recently (Table 4), thus, a recent, rapid pseudogenization of both duplicates seems the most probable explanation.

•Uncertain model for one pair. In the ABCC52-ABCC54 pair, one descendant (ABCA52) has a high expression level similar to that inferred for the ancestral state, and the other descendant (ABCA54) showed a specific expression pattern. Given the relatively high age of the duplication (high Ks), this pair seems unlikely to be involved in a slow, step-wise subfunctionalization because expression loss of te one copy is expected to be a rapid process. Since only two sites have been detected under positive selection, the possibility that this pair underwent a coding-level neofunctionalization process is also seemingly low. A gradual pseudogenization process that led to the step-wise loss of expression in one copy was possible, where such loss would have to be relatively recent due to the low Ka/Ks values of this pair. However, the lack of certainty in regard with their ancestral state further made the situation less clear. Therefore, we thought it is hard to suggest one best model for the ABCC52-ABCC54 pair.

Network asymmetry in which one gene in a pair acquired more co-expressed genes than the other (Additional file 6) could be identified in many tandem duplicate pairs in the seven sets of genes, indicating that each of the two duplicate genes could be involved in a different pathway or biological process. A similar phenomenon also has been reported in yeast [71]. Finally, six genes were found to be up-regulated under stress from DDT and one by exposure to TBT (unpublished data). This of suggests that there may be more duplicates that have been preserved in the genome through neofunctionalization or dosage balance.

## Conclusion

*T. thermophila *possesses a total of 165 ABC transporter genes, the largest number in this superfamily reported for any organism to date. The reason for *Tetrahymena *having so many ABC transporters is probably related to its lifestyle as a highly dispersed, free-living unicellular organism. For multicellular organisms and parasites, the cells are in more stable environments, whereas *Tetrahymena *must survive exposure to all the substances and organisms in the fresh water that surrounds it, and thus would benefit from having a large number of diverse genes to enable it to sense and respond to this correspondingly diverse and ever-changing set of environmental conditions. There is evidence that these genes have undergone lineage-specific expansions [27], creating a large, diverse complement of ABC transporters that could be involved in the efficient efflux of numerous endogenously produced toxins or elimination of a broad range of xenobiotics. The phylogenetic analyses, characterization of intron positions, and ortholog comparisons performed in the present study facilitated both the identification of progenitor genes and formation of strong hypotheses to explain the evolutionary origin and expansion of the ABC transporter superfamily in *Tetrahymena*. Our analyses indicated that the fusion of domains or half transporter genes played a role in the evolution of the diversity of ABC transporters in *Tetrahymena *along with gene expansion by duplication. These duplicates appear to have experienced subsequent divergence by accumulation of mutations and differences in expression patterns. Multiple different mechanisms including neofunctionalization, subfunctionalization, pseudogenization and dosage balance appear to account for this functional divergence.

## Methods

### Identification of genes

To identify ABC transporter genes in *Tetrahymena*, we first searched for predicted ABC transporters genes in the TIGR database (ftp://ftp.tigr.org/pub/data/Eukaryotic_Projects/t_thermophila/Transporter/) and extracted the 160 genes annotated as ABC transporters. These ABC transporters were divided into full transporters and half transporters, which were aligned separately to identify sequences of NBDs. The NBD sequences then were used to perform a BlastP search (default setting) in TGD [29] to identify other genes with an NBD-like sequence. A total of 185 genes were found, including 1 homolog of Rad50, 4 in the SMC family, 7 in the Muts family, 9 with partial NBD sequences, and 164 with complete NBDs. The DNA sequences of these 164 genes were used to do a BlastN search of the NCBI EST database and TBestDB (http://amoebidia.bcm.umontreal.ca/pepdb/searches/login.php), and the EST information of every gene was examined to determine whether there were any incorrectly predicted genes. RT-PCR was carried out to verify the cDNA sequences, and as a result, one incorrectly predicted gene was separated into two genes, giving a total of 165 predicted ABC transporter genes with complete NBDs. The protein sequences of these 165 predicted genes were used to perform BlastP searches in the NCBI non-redundant protein database, and hit scores and E-values were sufficient to sort the majority of the ABC transporter genes into 8 families (several sophisticated genes having to be determined by constructing a phylogenetic tree) [32]. The nomenclature for human ABC families (http://www.genenames.org/genefamily/abc.html) was used to name all ABC transporter genes in *Tetrahymena*. Orthologs with genes in *Paramecium *were detected as the reciprocal best hits in the Blast search, using all the 165 *T. thermophila *ABC genes as the query.

### Sequence alignment, phylogenetic analysis, and intron mapping

Owing to the difference in length of half and full ABC transporters, we used the NBDs for phylogenetic analysis of ABC transporters. All NBDs were sorted into the following three types: 1) NBDs of half transporters were given the same name as the gene (e.g. ABCA29); 2) N-terminal NBDs from full transporters were given their name of their gene followed by .1 (e.g. ABCA1.1); and 3) C-terminal NBDs from full transporters were given the name of their gene followed by .2 (e.g. ABCA1.2). The protein sequences of all NBDs were aligned using MAFFT (http://align.bmr.kyushu-u.ac.jp/mafft/online/server/index.html) and the matrix Blosum45. Then, the alignment was trimmed using the Trimal program [72] with the option -gt 0.1, and a maximum likelihood phylogenetic tree was constructed using PhyML [73] with 100 bootstrap iterations.

All nucleotide sequences of NBDs were used to map introns. First, each ABC transporter cDNA sequence was aligned with the scaffold sequence to find the position of the introns. Second, the alignment of NBD protein sequences was back-translated into a DNA alignment (codon alignment) of NBDs using RevTrans (http://www.cbs.dtu.dk/services/RevTrans/). Finally, the introns in the NBD regions were mapped manually into the codon alignment of NBD DNA sequences.

### Microarray data

The microarray data were retrieved from the *Tetrahymena *Gene Expression Database (TGED, http://tged.ihb.ac.cn/). This included the normalized microarray data published by in Miao *et al *[28] in which the microarray data of each gene consists of an expression profile containing 20 time points from three stages in the life cycle of *T*. *thermophila*, including 3 time-dependent points of growth, 7 time-dependent points of starvation, and 10 time-dependent points of conjugation. The heat map of the ABC transporter genes was calculated using the program ArrayStar version 2.0 (DNASTAR, Inc, Madison, WI).

### *Tetrahymena *strains, culture conditions, and toxicity experiments

The wild-type strain SB210 of *T*. *thermophila *was provided by Dr. E. Orias, University of California, Santa Barbara, and strain CU428 was provided by Dr. P.J. Bruns, Cornell University, Ithaca, NY. Cells were grown in SPP medium at 30°C [74]. Dr. Y. Xu, Institute of Hydrobiology, Chinese Academy of Sciences, China provided TCDD, which was dissolved in DMSO (Sigma) for use; β-estradiol was purchased from Sigma. Preparation of DDT was performed as previously described [75]. Aliquots from stock solutions of TCDD, DDT, and β-estradiol were added to culture medium to produce final concentrations of 1 ppb, 4 ppm, and 1 ppb. Cells from stock cultures of CU428 in early stationary phase (~2×10^5 ^cells/ml) were inoculated into TCDD, DDT, or β-estradiol-treated media and incubated for 24 hours at 30°C. The same volume of DMSO used in treatment cultures also was included in cultures used as negative controls.

### Total RNA isolation, cDNA synthesis and real-time PCR

Total RNA was isolated using the RNeasy Plus Mini Kit (Qiagen) after cells were homogenized with QIAshredder (Qiagen) according to the manufacturer's instructions. RNA integrity was verified using a Bioanalyzer 2100 (Agilent). RNA samples were treated with DNase (Promega) and subsequently were reverse transcribed into double stranded cDNA using M-MLV reverse transcriptase RNase H^+ ^(TOYOBO). Real-time PCR reactions and calculation of the relative expression levels of *ABCB15 *and *ABCG25 *were the same as previously described [76].

### Analyzing evolution of gene expression and inferring ancestral state of expression

The procedure used for analyzing evolution of gene expression followed the steps described in [66]. First, the pairwise similarity was calculated using FASTA (http://fasta.bioch.virginia.edu/fasta_www2/fasta_www.cgi). Then, the following two criteria described in [66] were used to partition the superfamily of ABC transporters: 1) sequences must contain a FASTA-alignable region that comprises greater than 80% of the longer protein; 2) two genes resemble each other more than a specified threshold (35%). Based on these criteria, 7 sets of genes were identified that were suitable for analysis of the evolution of gene expression, including 2 sets each in the ABCA, ABCB, and ABCC families and 1 set in the ABCG family. Protein sequences of each set of genes were aligned using MAFFT. Then the alignments were back translated into the codon alignment of DNA sequences using RevTrans.

The GTR+I+G model of evolution was selected by ModelTest [77], and ML trees were constructed by PAUP 4.0b10 [78]. The microarray data of each gene from 20 points in time were log2-base transformed and represented the character data of taxa tips. Then, both the phylogenetic trees and the log-transformed microarray data were input as a set of continuous data into the program CoMET [79]. Using default settings, 9 maximum likelihood models of the evolution of gene expression were compared according to the Akaike Information Criterion (AIC), and the model with the minimum AIC value was chosen as the best fitting model of evolution of gene expression.

To estimate the best fitting model of the evolution of duplicated ABC genes, the expression pattern of each of the 7 sets of genes was characterized according to the following system of four types based on the heat map of expression over 20 time points in the life cycle of *T. thermophila*: 0, silent; 1, low expression (some points may silent); 2, specific high expression (in terms of point or stage); 3, continuous high expression. The ancestral expression states were constructed by parsimony and likelihood methods with the program Mesquite [80] using the four types of expression states (0, 1, 2 and 3) as characters of taxa.

### PCC calculation, searching for candidate co-expressed genes, and detection of the positive selection

Information on the co-expression of genes of *T. thermophila *was found in TGED. The Pearson Correlation Coefficient (PCC) of a pair of genes was calculated using a C++-compiled program (available upon request). Candidates for co-expressed genes were identified by the PCC using a threshold of 0.8 (This threshold guaranteed including most genes in KEGG-predicted conserved pathways between *T. thermophila *and other eukaryotes). Every ABC transporter gene in the 7 sets was searched in TGED, and the candidates for co-expressed genes were obtained. To measure the difference in numbers of candidate co-expressed genes, we adopted the following two methods: 1) calculating the quotient (high number/low number) of candidate co-expressed genes between two genes in a pair and 2) calculating the difference (high number-low number) in candidate co-expressed genes between two genes in a pair. Finally, the percentage of pairs in which the quotient > 2 and the difference > 100 was calculated.

The set of 7 genes identified by the procedures given above were used to analyze evolutionary patterns of divergence in expression of genes. Their nucleotide sequences were aligned codon by codon, using the aligned protein sequences as guides, and an ML tree of each set was constructed using PhyML. Then, positive selection was tested by PAML using the branch (Model0), site (Nsites = 1, 2, 7 and 8) and branch-site (Model A, specifying the two tandem repeats as foreground lineage) models [81]. In addition, the non-synonymous substitution (Ka) and synonymous substitution (Ks) for gene pairs in each set also were calculated.

### Microarray data accession numbers

Microarray data have been deposited with the NCBI Gene Expression Omnibus (http://www.ncbi.nlm.nih.gov/geo) under accession numbers listed in document S11 in Miao *et al *[28] ... (doi:10.1371/journal.pone.0004429).

## Authors' contributions

WM, JX and CF conceived and designed the experiments. JX and LF analyzed the data and performed all experiments except the microarray. YD assisted in the experiments. WM performed the microarray experiments. WM, JX, LF and CF interpreted the data and wrote the paper. All authors have read and approved the final manuscript.

## Supplementary Material

Additional file 1 **Characterization of the 165 ABC genes of *T. thermophila***. Families were named according to the nomenclature of Human ABC transporters. Gene ID, structure, intron number, ESTs, protein length and scaffold are listed. ^a^: GST_N_Mu, GST_N family and Class Mu subfamily; ^b^: Peptidase_S9 domain, Prolyl oligopeptidase family; ^c^: Protein-L-isoaspartate (D-aspartate) O-methyltransferase (PCMT). Click here for file

Additional file 2 **Sketch of ABCB23**. The sequence shown here was amplified from cDNA using two primers that are highlighted in red and are matched to regions in NBD-1 and TMD-2. The continuous sequence between NBD-1 and TMD-2 confirmed as ABCB23 was one full transporter and not two half transporters. Click here for file

Additional file 3 **Real-time PCR analysis of expression of *ABCB15 *and *ABCG25 *in *T. thermophila *CU428 treated with DMSO, TCDD, DDT, and β-estradiol**. Real-time PCR reactions were performed in triplicate for each cDNA sample, and values were the means of three determinations. DSMO = Dimethyl Sulphoxide; TCDD = 2, 3, 7, 8, tetrachlorodibenzo-*p*-dioxin; DDT = dichlorodiphenyltrichloroethane. * *p *< 0.05. Click here for file

Additional file 4 **Intron structures of eukaryotic ABCE genes**. Each red vertical line represents an intron, a and b represent two ABCE genes in *Arabidopsis thaliana*. The blue panel represents the intron position conserved between *Tetrahymena *and *Arabidopsis *ABCE genes. Click here for file

Additional file 5 **Expression profiles of ABCD1 and ABCD2**. 20 time points of the three physiological/development stages of *Tetrahymena*. For growing cells, **L-l**, **L-m **and **L-h **correspond to ~1×10^5 ^cells/ml, ~3.5×10^5 ^cells/ml and ~1×10^6 ^cells/ml, respectively. For starvation, ~2×10^5 ^cells/ml were collected at intervals of 0, 3, 6, 9, 12, 15 and 24 hours (referred to as **S-0, S-3, S-6, S-9, S-12, S-15 **and **S-24**, respectively). For conjugation, equal volumes of B2086 and CU428 cells were mixed in culture, and samples were collected at intervals of 0, 2, 4, 6, 8, 10, 12, 14, 16 and 18 hours after mixing (referred to as **C-0, C-2, C-4, C-6, C-8, C-10, C-12, C-14, C-16 **and **C-18**, respectively). Click here for file

Additional file 6 **Different candidates for co-expression of genes between two genes in each pair**. The quotient (high number/low number) and the difference (high number/low number) of candidate co-expressed gene number between two genes in each pair have been calculated. ^a^: the percentage of pairs in which the quotient > 2. ^b^: the percentage of pairs in which the difference > 100. Click here for file
