# Supplementary material for: Genome-wide identification and evolution of ATP-binding cassette transporters in the ciliate Tetrahymena thermophila: A case of functional divergence in a multigene family
Source: BMC Evol Biol. 2010 Oct 27;10:330. doi: 10.1186/1471-2148-10-330 (PMC2984421; doi:10.1186/1471-2148-10-330)
Supplement: Additional file 1 — Characterization of the 165 ABC genes of T. thermophila. Families were named according to the nomenclature of Human ABC transporters. Gene ID, structure, intron number, ESTs, protein length and scaffold are listed. a: GST_N_Mu, GST_N family and Class Mu subfamily; b: Peptidase_S9 domain, Prolyl oligopeptidase family; c: Protein-L-isoaspartate (D-aspartate) O-methyltransferase (PCMT). [file 1471-2148-10-330-S1.DOC]

| **Gene Name** | **Gene ID** | **Structure** | **Intron number** | **EST** | **Length（bp）** | **Choromosome ID** | ***Paramecium* Ortholog** |
| --- | --- | --- | --- | --- | --- | --- | --- |
| **ABCA Family** |  |  |  |  |  |  |  |
| ABCA1 | TTHERM_00476990 | (TMD-NBD)_2_ | 4 | - | 1829 | CH445626 |  |
| ABCA2 | TTHERM_00476910 | (TMD-NBD)_2_ | 3 | DY679249 | 1836 | CH445626 |  |
| ABCA3 | TTHERM_00717640 | (TMD-NBD)_2_ | 13 | - | 2778 | CH445661 |  |
| ABCA4 | TTHERM_01417320 | (TMD-NBD)_2_ | 2 | - | 1787 | CH670451 |  |
| ABCA5 | TTHERM_00209390 | (TMD-NBD)_2_ | 5 | - | 1858 | CH445786 |  |
| ABCA6 | TTHERM_00138260 | (TMD-NBD)_2_ | No intron | DY679249 | 1776 | CH445601 |  |
| ABCA7 | TTHERM_00898300 | (TMD-NBD)_2_ | No intron | - | 1735 | CH445574 | GSPATP00013954001 |
| ABCA8 | TTHERM_00532790 | (TMD-NBD)_2_ | 16 | CX591747  CX586768  EC271472  CX573728  DY679250  CX592086  DY679249 | 2049 | CH445539 |  |
| ABCA9 | TTHERM_00011470 | (TMD-NBD)_2_ | 13 | CX589330 | 1715 | CH445775 |  |
| ABCA10 | TTHERM_00693300 | (TMD-NBD)_2_ | 6 | - | 1800 | CH445549 |  |
| ABCA11 | TTHERM_00693290 | (TMD-NBD)_2_ | 6 | DY679249 | 1761 | CH445549 |  |
| ABCA12-1 | TTHERM_00694300 | (TMD-NBD)_2_ | / | - | / | CH445549 |  |
| ABCA12-2 |  | (TMD-NBD)_2_ | / | - | / |  |  |
| ABCA13 | TTHERM_00693280 | (TMD-NBD)_2_ | 6 | - | 1738 | CH445549 |  |
| ABCA14 | TTHERM_00694310 | (TMD-NBD)_2_ | 6 | CX574943 | 1701 | CH445549 |  |
| ABCA15 | TTHERM_00301860 | (TMD-NBD)_2_ | 7 | - | 1749 | CH445536 | GSPATP00017796001 |
| ABCA16 | TTHERM_00301870 | (TMD-NBD)_2_ | 6 | - | 1733 | CH445536 |  |
| ABCA17 | TTHERM_00919560 | (TMD-NBD)_2_ | 5 | DY677415  DY677414 | 1744 | CH445400 | GSPATP00022092001 |
| ABCA18 | TTHERM_00919550 | (TMD-NBD)_2_ | 7 | CX580100 | 1940 | CH445400 |  |
| ABCA19 | TTHERM_00323060 | (TMD-NBD)_2_ | 8 | CX578847  EC269003  EC268948  CX577837  CX585811 | 1147 | CH445692 |  |
| ABCA20 | TTHERM_00323080 | (TMD-NBD)_2_ | 12 | CX578847  EC269003  CX577837  CX585811 | 1342 | CH445692 | GSPATP00000078001 |
| ABCA21 | TTHERM_00218430 | (TMD-NBD)_2_ | 8 | CX590861 | 1186 | CH445588 |  |
| ABCA22 | TTHERM_00862740 | (TMD-NBD)_2_ | 11 | CX590861 | 1407 | CH445638 |  |
| ABCA23 | TTHERM_00529470 | (TMD-NBD)_2_ | 11 | CX576236  CX583458  CX585811 | 1322 | CH670351 |  |
| ABCA24 | TTHERM_00635770 | (TMD-NBD)_2_ | 13 | - | 1402 | CH445744 |  |
| ABCA25 | TTHERM_00146350 | (TMD-NBD)_2_ | 13 | - | 1412 | CH445731 |  |
| ABCA26 | TTHERM_00649360 | (TMD-NBD)_2_ | 10 | CX591235  EC269148  BF845507  EC269147  CX578285 | 1295 | CH670355 |  |
| ABCA27 | TTHERM_00125090 | (TMD-NBD)_2_ | 11 | EC271624  EC273936  DY679111  EC271623  EC273935  DY679100 | 1298 | CH445650 |  |
| ABCA28 | TTHERM_00535970 | (TMD-NBD)_2_ | 9 | CX590861  EC269147 | 1346 | CH445551 |  |
| ABCA29 | TTHERM_00188440 | TMD-NBD | 4 (3) | - | 1062 | CH445644 |  |
| ABCA30 | TTHERM_00728910 | TMD-NBD | 9 | DY676887  DY676886 | 953 | CH445559 | GSPATP00007250001 |
| ABCA31 | TTHERM_00047720 | TMD-NBD | 2 | - | 1069 | CH445663 |  |
| ABCA32 | TTHERM_00189100 | TMD-NBD | 10 | DY683805  CX592008 | 932 | CH445644 | GSPATP00005865001 |
| **ABCB Family** |  |  |  |  |  |  |  |
| ABCB1 | TTHERM_00550900 | TMD-NBD | 0 | CX582511 | 698 | CH445762 | GSPATP00022014001 |
| ABCB2 | TTHERM_00550890 | TMD-NBD | 0 | CX582511 | 701 | CH445762 |  |
| ABCB3 | TTHERM_00420410 | TMD-NBD | 2 | CX579610  EC269710 | 655 | CH670347 | GSPATP00023474001 |
| ABCB4 | TTHERM_00463500 | TMD-NBD | 9 | CX586507 | 937 | CH445612 |  |
| ABCB5 | TTHERM_00070900 | TMD-NBD | 1 | - | 723 | CH445782 |  |
| ABCB6 | TTHERM_00136000 | TMD-NBD | 0 | - | 844 | CH445601 |  |
| ABCB7 | TTHERM_00415580 | TMD-NBD | 3 | - | 1439 | CH445785 |  |
| ABCB8 | TTHERM_00037430 | TMD-NBD | 1 | - | 714 | CH445789 |  |
| ABCB9 | TTHERM_00823540 | TMD-NBD | 0 | - | 688 | CH670366 |  |
| ABCB10 | TTHERM_00541510 | TMD-NBD | 3 | - | 600 | CH445792 |  |
| ABCB11 | TTHERM_00541500 | TMD-NBD | 1 | - | 1114 | CH445792 |  |
| ABCB12 | TTHERM_00684430 | TMD-NBD | 7 | - | 542 | CH445530 | GSPATP00036464001 |
| ABCB13 | TTHERM_01084320 | TMD-NBD | 0 | CX576982 | 663 | CH670393 | GSPATP00014680001 |
| ABCB14 | TTHERM_00083790 | (TMD-NBD)_2_ | 9 | - | 1313 | CH445695 |  |
| ABCB15 | TTHERM_00240450 | (TMD-NBD)_2_ | 9 | - | 1334 | CH445533 |  |
| ABCB16 | TTHERM_00241480 | (TMD-NBD)_2_ | 11 | - | 1334 | CH445533 |  |
| ABCB17 | TTHERM_01151440 | (TMD-NBD)_2_ | 4 | - | 1338 | CH670406 | GSPATP00032924001 |
| ABCB18 | TTHERM_01151450 | (TMD-NBD)_2_ | 2 | - | 1306 | CH670406 |  |
| ABCB19 | TTHERM_00502630 | (TMD-NBD)_2_ | 6 | - | 1295 | CH445565 |  |
| ABCB20 | TTHERM_00502619 | (TMD-NBD)_2_ | 5 | 2 | 1317 | CH445565 |  |
| ABCB21 | TTHERM_00423390 | (TMD-NBD)_2_ | 4 | CX591480  DY676730 | 1289 | CH445674 |  |
| ABCB22 | TTHERM_00685900 | (TMD-NBD)_2_ | 7 | - | 1318 | CH445530 |  |
| ABCB23 | TTHERM_00803570 | (TMD-NBD)_2_ | 16 | CX579187  CX579281  CX579045  DY682305 | 2105 | CH445705 |  |
| ABCB24 | TTHERM_00414540 | (TMD-NBD)_2_ | 6 | - | 1354 | CH445785 |  |
| ABCB25 | TTHERM_00423380 | (TMD-NBD)_2_ | 9 | CX591480  DY676730  DY676730  CX591480 | 1300 | CH445674 |  |
| ABCB26 | TTHERM_00423389 | (TMD-NBD)_2_ | 4 | - | 1302 | CH445674 |  |
| **ABCC Family** |  |  |  |  |  |  |  |
| ABCC1 | TTHERM_00414290 | (TMD-NBD)_2_ | 6 | CX573440 | 1448 | CH445785 |  |
| ABCC2 | TTHERM_00344220 | (TMD-NBD)_2_ | 6 | - | 1324 | CH445615 |  |
| ABCC3 | TTHERM_00964340 | (TMD-NBD)_2_ | 14 | CX579396 | 1646 | CH445768 |  |
| ABCC4 | TTHERM_00951710 | (TMD-NBD)_2_ | 8 | CX579396 | 1330 | CH445557 |  |
| ABCC5 | TTHERM_00951770 | (TMD-NBD)_2_ | 5 | - | 1501 | CH445557 |  |
| ABCC6 | TTHERM_00951730 | (TMD-NBD)_2_ | 5 | CX579396 | 1494 | CH445557 |  |
| ABCC7 | TTHERM_00942770 | (TMD-NBD)_2_ | 5 | CX579396 | 1498 | CH670374 |  |
| ABCC8 | TTHERM_01227870 | (TMD-NBD)_2_ | 5 | - | 1472 | CH445682 |  |
| ABCC9 | TTHERM_00951800 | (TMD-NBD)_2_ | 6 | CX588543 | 1497 | CH445557 |  |
| ABCC10 | TTHERM_00320020 | (TMD-NBD)_2_ | 6 | CX587102 | 1428 | CH445692 | GSPATP00008732001 |
| ABCC11 | TTHERM_00320030 | (TMD-NBD)_2_ | 9 | - | 1340 | CH445692 |  |
| ABCC12 | TTHERM_00346710 | (TMD-NBD)_2_ | 11 | - | 1292 | CH445615 |  |
| ABCC13 | TTHERM_00526180 | (TMD-NBD)_2_ | 6 | CX586693  DY683881 | 958 | CH445398 |  |
| ABCC14 | TTHERM_00346400 | (TMD-NBD)_2_ | 6 | - | 1399 | CH445615 |  |
| ABCC15 | TTHERM_00344080 | (TMD-NBD)_2_ | 10 | - | 1262 | CH445615 |  |
| ABCC16 | TTHERM_00594090 | (TMD-NBD)_2_ | 9 | - | 1328 | CH445720 |  |
| ABCC17 | TTHERM_00160950 | (TMD-NBD)_2_ | 5 | - | 1159 | CH445754 |  |
| ABCC18 | TTHERM_00558230 | (TMD-NBD)_2_ | 13 | - | 1551 | CH445564 |  |
| ABCC19 | TTHERM_00558220 | (TMD-NBD)_2_ | 16 | - | 1740 | CH445564 |  |
| ABCC20 | TTHERM_00137850 | (TMD-NBD)_2_ | 17 | EC272946  EC272947 | 1423 | CH445601 |  |
| ABCC21 | TTHERM_00006240 | (TMD-NBD)_2_ | 12 | EC270362  EC270361  DY676376 | 1233 | CH445775 |  |
| ABCC22 | TTHERM_00219530 | (TMD-NBD)_2_ | 14 | - | 1550 | CH445588 |  |
| ABCC23 | TTHERM_00616180 | (TMD-NBD)_2_ | 13 | - | 1478 | CH445535 |  |
| ABCC24 | TTHERM_00912240 | (TMD-NBD)_2_ | 11 | - | 1343 | CH445597 | GSPATP00008991001 |
| ABCC25 | TTHERM_00912250 | (TMD-NBD)_2_ | 13 | - | 1256 | CH445597 |  |
| ABCC26 | TTHERM_00912270 | (TMD-NBD)_2_ | 13 | - | 1288 | CH445597 |  |
| ABCC27 | TTHERM_00912260 | (TMD-NBD)_2_ | 14 | - | 1233 | CH445597 |  |
| ABCC28 | TTHERM_00706360 | (TMD-NBD)_2_ | 12 | - | 1292 | CH445732 |  |
| ABCC29 | TTHERM_00561570 | (TMD-NBD)_2_ | 2 | - | 1384 | CH445743 |  |
| ABCC30 | TTHERM_00561590 | (TMD-NBD)_2_ | 3 | - | 1353 | CH445743 |  |
| ABCC31 | TTHERM_01358410 | (TMD-NBD)_2_ | 7 | CX579960  CX575921  CX582321  CX587863  CX588040  CX582772  CX586555  CX588067  CX576165  CX585936  CX587192  CX590814  CX580015  CX578556  CX583026  CX578778  CX575356  DY684508  CX588328  CX571785  CX579002  DY684027  CX572663  DY682328 | 1605 | CH445678 |  |
| ABCC32 | TTHERM_00584900 | (TMD-NBD)_2_ | 5 | - | 1357 | CH670352 |  |
| ABCC33 | TTHERM_00683350 | (TMD-NBD)_2_ | 5 | CX573802 | 1444 | CH445417 |  |
| ABCC34 | TTHERM_00161860 | (TMD-NBD)_2_ | 6 | - | 1451 | CH445754 |  |
| ABCC35 | TTHERM_01194710 | (TMD-NBD)_2_ | 7 | - | 1366 | CH670412 |  |
| ABCC36 | TTHERM_00785880 | (TMD-NBD)_2_ | 5 | CX572081 | 1489 | CH445566 |  |
| ABCC37 | TTHERM_00440640 | (TMD-NBD)_2_ | 7 | - | 1425 | CH445623 |  |
| ABCC38 | TTHERM_00440660 | (TMD-NBD)_2_ | 7 | - | 1420 | CH445623 |  |
| ABCC39 | TTHERM_00283160 | (TMD-NBD)_2_ | 7 | CX587368  DY683356  CX576067 | 1409 | CH445618 |  |
| ABCC40 | TTHERM_00479080 | (TMD-NBD)_2_ | 5 | CX588962 | 1496 | CH445626 | GSPATP00008808001 |
| ABCC41 | TTHERM_00998800 | (TMD-NBD)_2_ | 5 | - | 1480 | CH670377 |  |
| ABCC42 | TTHERM_00916430 | (TMD-NBD)_2_ | 8 | CX586702 | 1470 | CH445597 |  |
| ABCC43 | TTHERM_00592990 | (TMD-NBD)_2_ | 9 | - | 1341 | CH445720 |  |
| ABCC44 | TTHERM_00593020 | (TMD-NBD)_2_ | 7 | - | 1307 | CH445720 |  |
| ABCC45 | TTHERM_00593010 | (TMD-NBD)_2_ | 8 | - | 1247 | CH445720 |  |
| ABCC46 | TTHERM_01009850 | (TMD-NBD)_2_ | 7 | - | 1313 | CH445624 |  |
| ABCC47 | TTHERM_00574250 | (TMD-NBD)_2_ | 8 | EC271670 | 1361 | CH445759 |  |
| ABCC48 | TTHERM_00630580 | (TMD-NBD)_2_ | 8 | EC271670  CX573859  CX577639 | 1260 | CH445558 |  |
| ABCC49 | TTHERM_00630560 | (TMD-NBD)_2_ | 7 | CX573859  EC271670 | 1310 | CH445558 |  |
| ABCC50 | TTHERM_00265150 | (TMD-NBD)_2_ | 7 | CX577639  CX578954  CX587368  DY683356 | 1315 | CH445763 |  |
| ABCC51 | TTHERM_00024080 | (TMD-NBD)_2_ | 8 | CX587368  DY683356 | 1439 | CH445775 |  |
| ABCC52 | TTHERM_00661580 | (TMD-NBD)_2_-GST_Mu^a^ | 4 | - | 1643 | CH445598 |  |
| ABCC53 | TTHERM_00624760 | (TMD-NBD)_2_ | 3 | - | 1411 | CH445561 |  |
| ABCC54 | TTHERM_00661590 | (TMD-NBD)_2_ | 6 | - | 1298 | CH445598 |  |
| ABCC55 | TTHERM_01083010 | (TMD-NBD)_2_ | 8 | - | 1235 | CH670392 | GSPATP00001809001 |
| ABCC56 | TTHERM_00522440 | (TMD-NBD)_2_ | 4 | - | 1457 | CH445667 |  |
| ABCC57 | TTHERM_00372620 | (TMD-NBD)_2_ | 17 | CX572081 | 3035 | CH445755 |  |
| ABCC58 | TTHERM_00300370 | (TMD-NBD)_2_ | 9 | - | 2102 | CH445536 |  |
| ABCC59 | TTHERM_00243670 | (TMD-NBD)_2_- P_S9^b^ | 17 | CX579799 | 1868 | CH445546 |  |
| ABCC60 | TTHERM_00313120 | (TMD-NBD)_2_ | 18 | - | 1072 | CH670346 |  |
| **ABCD Family** |  |  |  |  |  |  |  |
| ABCD1 | TTHERM_00836750 | TMD-NBD | 9 | BM396220 | 719 | CH445529 | GSPATP00024858001 |
| ABCD2 | TTHERM_00564120 | TMD-NBD | 7 | - | 694 | CH445568 |  |
| **ABCE Family** |  |  |  |  |  |  |  |
| ABCE1 | TTHERM_00850570 | NBD-NBD | 6 | - | 617 | CH445607 | GSPATP00016840001 |
| **ABCF Family** |  |  |  |  |  |  |  |
| ABCF1 | TTHERM_00313119 | NBD-NBD | 6 | - | 725 | CH670346 | GSPATP00025569001 |
| ABCF2 | TTHERM_01014620 | NBD-NBD | No intron | BM396561  BM398557 | 571 | CH670380 |  |
| **ABCG Family** |  |  |  |  |  |  |  |
| ABCG1 | TTHERM_00654020 | NBD-TMD | 1 | - | 598 | CH445670 |  |
| ABCG2 | TTHERM_00418430 | NBD-TMD | 1 | - | 607 | CH445785 |  |
| ABCG3 | TTHERM_00035440 | NBD-TMD | 1 | - | 607 | CH445789 |  |
| ABCG4 | TTHERM_00035420 | NBD-TMD | 3 | - | 566 | CH445789 |  |
| ABCG5 | TTHERM_00035430 | NBD-TMD | 2 | - | 592 | CH445789 |  |
| ABCG6 | TTHERM_00035400 | NBD-TMD | 3 | - | 553 | CH445789 |  |
| ABCG7 | TTHERM_00032890 | NBD-TMD | 5 | - | 607 | CH445789 |  |
| ABCG8 | TTHERM_00033900 | NBD-TMD | 5 | - | 610 | CH445789 |  |
| ABCG9 | TTHERM_00034910 | NBD-TMD | 5 | - | 611 | CH445789 |  |
| ABCG10 | TTHERM_00032880 | NBD-TMD | 6 | CX573444 | 582 | CH445789 |  |
| ABCG11 | TTHERM_00600070 | NBD-TMD | 3 | - | 585 | CH670353 |  |
| ABCG12 | TTHERM_00031790 | NBD-TMD | 3 | CX584647  CX583637 | 599 | CH445789 |  |
| ABCG13 | TTHERM_00031770 | NBD-TMD | 5 | - | 575 | CH445789 |  |
| ABCG14 | TTHERM_00031800 | NBD-TMD | 3 | CX584647 | 601 | CH445789 | GSPATP00018850001 |
| ABCG15 | TTHERM_00031810 | NBD-TMD | 3 | - | 593 | CH445789 |  |
| ABCG16 | TTHERM_00031820 | NBD-TMD | 3 | - | 599 | CH445789 |  |
| ABCG17 | TTHERM_00031830 | NBD-TMD | 2 | CX584647 | 601 | CH44578 |  |
| ABCG18 | TTHERM_00034940 | NBD-TMD | 7 | - | 564 | CH445789 |  |
| ABCG19 | TTHERM_00034920 | NBD-TMD | 6 | - | 604 | CH445789 |  |
| ABCG20 | TTHERM_00035320 | NBD-TMD | 1 | - | 627 | CH445789 | GSPATP00018406001 |
| ABCG21 | TTHERM_00035300 | NBD-TMD | No intron | - | 600 | CH445789 |  |
| ABCG22 | TTHERM_00035360 | NBD-TMD | No intron | - | 600 | CH445789 |  |
| ABCG23 | TTHERM_00035280 | NBD-TMD | No intron | - | 585 | CH445789 |  |
| ABCG24 | TTHERM_00035270 | NBD-TMD | 2 | - | 565 | CH445789 |  |
| ABCG25 | TTHERM_00035330 | NBD-TMD | No intron | - | 593 | CH445789 |  |
| ABCG26 | TTHERM_00035380 | NBD-TMD | No intron | - | 598 | CH445789 |  |
| ABCG27 | TTHERM_00035350 | NBD-TMD | No intron | - | 637 | CH445789 |  |
| ABCG28 | TTHERM_00409090 | NBD-TMD | 2 | - | 532 | CH445583 |  |
| ABCG29 | TTHERM_00624590 | NBD-TMD | 2 | - | 614 | CH445561 |  |
| ABCG30 | TTHERM_01128600 | NBD-TMD | 4 | - | 551 | CH445455 |  |
| ABCG31 | TTHERM_01002640 | NBD-TMD | 5 | - | 659 | CH670378 |  |
| ABCG32 | TTHERM_01002630 | NBD-TMD | 4 | - | 438 | CH670378 |  |
| ABCG33 | TTHERM_00713380 | NBD-TMD | 6 | - | 867 | CH445482 | GSPATP00000757001 |
| ABCG34 | TTHERM_00794030 | NBD-TMD | 4 | - | 459 | CH445581 |  |
| ABCG35 | TTHERM_00580360 | NBD-TMD-PCMT^c^ | 5 | CX585155 | 1256 | CH445556 |  |
| ABCG36 | TTHERM_00561600 | NBD-TMD | 22 | - | 4926 | CH445743 |  |
| ABCG37 | TTHERM_00032870 | (NBD-TMD)_2_ | 8 | CX584647 | 1129 | CH445789 |  |
| ABCG38 | TTHERM_00033910 | (NBD-TMD)_2_ | 23 | - | 1497 | CH445789 |  |
| ABCG39 | TTHERM_00035390 | (TMD-NBD)_2_ | 4 | - | 1256 | CH445789 |  |
| **ABCH Family** |  |  |  |  |  |  |  |
| ABCH1 | TTHERM_00522300 | NBD | 4 | - | 408 | CH445667 | GSPATP00008414001 |
| ABCH2 | TTHERM_00118680 | NBD | 6 | DY684117 | 355 | CH445735 | GSPATP00022829001 |
