# Supplementary material for: Genome-wide identification and evolution of ATP-binding cassette transporters in the ciliate Tetrahymena thermophila: A case of functional divergence in a multigene family
Source: BMC Evol Biol. 2010 Oct 27;10:330. doi: 10.1186/1471-2148-10-330 (PMC2984421; doi:10.1186/1471-2148-10-330)
Supplement: Additional file 2 — Sketch of ABCB23. The sequence shown here was amplified from cDNA using two primers that are highlighted in red and are matched to regions in NBD-1 and TMD-2. The continuous sequence between NBD-1 and TMD-2 confirmed as ABCB23 was one full transporter and not two half transporters. [file 1471-2148-10-330-S2.PDF]

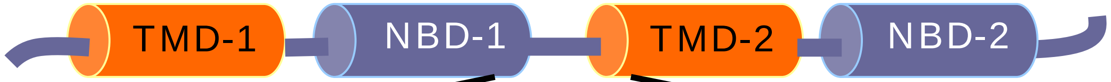

CATCAATCTCAAATGGCTACGTTCTTAATTTGGATACATAGGATAAGAACCAGAAATAT
TCTTTGGAAGCATTAAGAGTAATTTGCTCATTTCTAAACGGGAAGCATCTTTAGAAGAG
TTGTATAATGTACTAGAATAAGTTTATTTATTGAATATTGTGTAAAATTTAGACCTTGGT
ATAGATTCTGATGTTAAAAATTTGTCAGTTGGAGAGAAACAAAGATTGTGTATTGCTCG
TGTACTGTTGAGAAAACCAAAAATATTTATTTTTGATGAACCTATTTCTTCTTAGGATTT
TAAAATGGAAAAATAAATACAATAACTAATTGATGAAATAACGAAAAATTATACATCG
ATAATCATATCTCACTCTTTTAGTAAAATCTAACAATAAGATAAAATTATTATAATGAA
CAAAGGTAAAATATTGAAGTAAGGAAAGCACTCAGATTTAATTAATGAAGATGGAATA
TATCTTAACATAATTTAAGAGCGCTAATATATTACTGAAGAAATTGACTACTTTCTGAA
ACAATAAATAGAAGAACAAATGAAAAAAACACTTGAATTTGAAGAAGTTGAATCCCTT
AATAACTAAGATATTGATTCAAATCATTTAAATAAATTTTAAGAAATTAAATTATCAAA
AAGCGAATCTGTTAAAAAGCTTGATAAACAAAAAAAAACAATTAGAAAGATTAAAAAGC
AAAATTTAGTATAACATTGATAAAAAATTTATGAATATTGATTTTGCTTATCAAAATAAT
GAGTTGCCTCCTGAAAATGTGTTATTTAACTTTGGAGAACATAAAACATTTAGTTAATTT
ACCAATCATTAAAAAAAAGAGAAGAAGCACTCTAATTTAGAGTAACAATTAAGTTTAATT
AATCTCAAATAGTGTAAACTAAAAAGATAATTGTGTAGTTAGTAGCTAAGGACAAGTTT
CTAAAAAGTCTAATTCTTAGGAATAAAAAAAGGCTTACTTGCTTGATGCTTAAGAAATA
GAATTACAATTAATATCAGGTTAAATTGACCATAAAAAAATTTAGTAAATTGAGAGTAT
ATAACAACAACAAAATTCAGTTCTTAGAGAAGAAGAAATAAAAGCAAGCTAGGACATG
AACTCTGATTTAAAGTCCCAAACTTCTTAGTTAAATGTGAATGATAGTAAAAAAAGATA
AGATATGTACGGTAACGGAAAGCAATGGTTGATAAACTACTAAAATTAAGTTAGGTATA
ATACATTAACAATTTTTAAACATATCTTAAAAGAAAAAGTGGCTTTGAAAGGAATGATA
TGGTAAACTATAGGCATATTGATGGCTATTATTCATGGAGCAACTTATCCCTTTAATTGC
ATAATTATAGCTGATACAATTTATAATGTTTCTAATTACACCCTCAACCCAACTTTGAAA
AACTAAAATCTTATTATTTAAAGTCTGATCAAATTTATTTATACAGGAATTACGGGTTTA
ATTTCTGCTACTCTTATGACTTATTCTTTAAATGTTGCAGGAGAAACGCTTTCTTTCAAT
CTCAAA
