# Supplementary figures and images for: Genome-wide identification and evolution of ATP-binding cassette transporters in the ciliate Tetrahymena thermophila: A case of functional divergence in a multigene family
Source: BMC Evol Biol. 2010 Oct 27;10:330. doi: 10.1186/1471-2148-10-330 (PMC2984421; doi:10.1186/1471-2148-10-330)

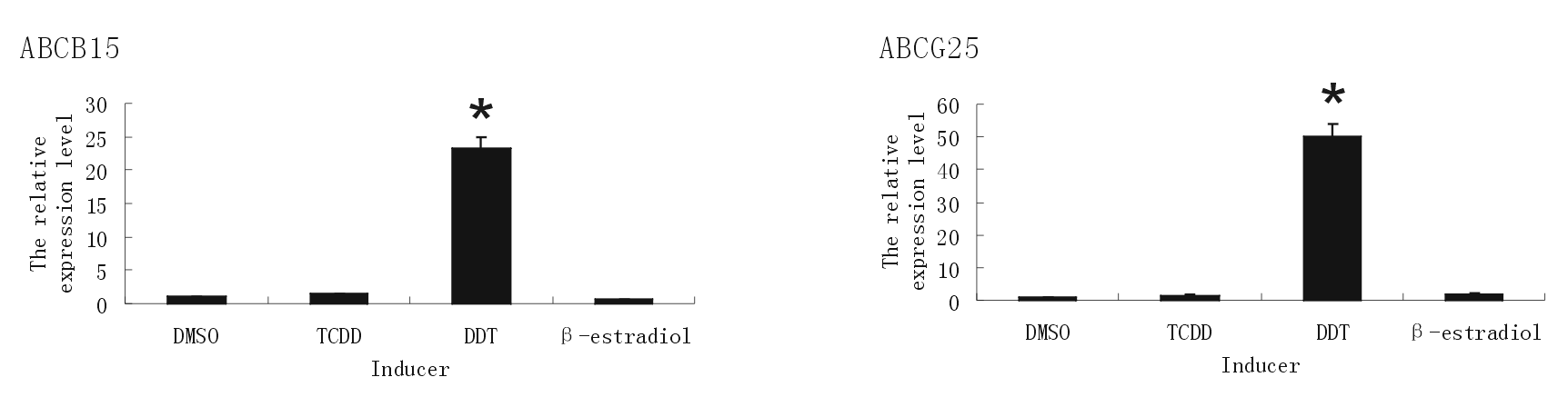

Supplement: Additional file 3 — Real-time PCR analysis of expression of ABCB15 and ABCG25 in T. thermophila CU428 treated with DMSO, TCDD, DDT, and β-estradiol. Real-time PCR reactions were performed in triplicate for each cDNA sample, and values were the means of three determinations. DSMO = Dimethyl Sulphoxide; TCDD = 2, 3, 7, 8, tetrachlorodibenzo-p-dioxin; DDT = dichlorodiphenyltrichloroethane. * p < 0.05. [file 1471-2148-10-330-S3.TIFF]

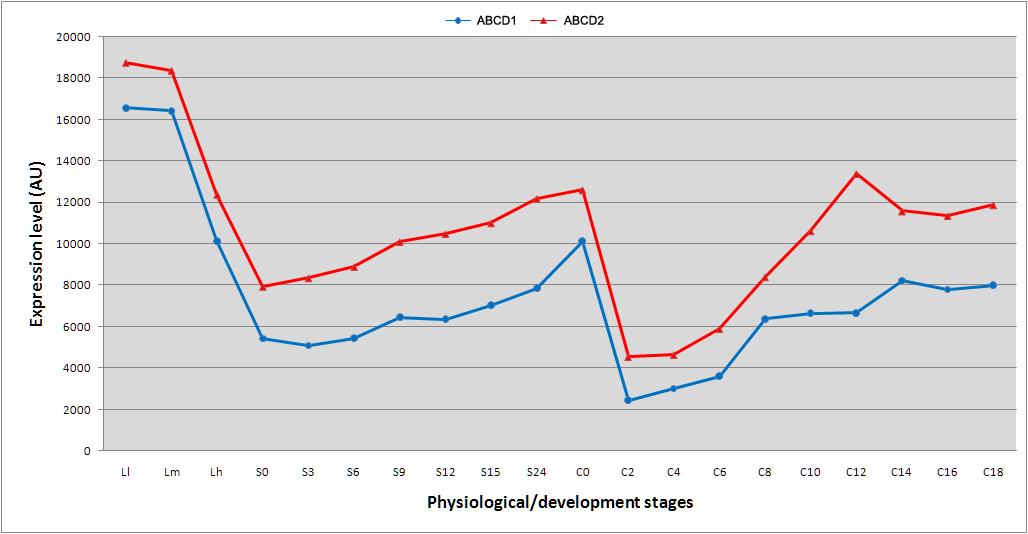

Supplement: Additional file 4 — Intron structures of eukaryotic ABCE genes. Each red vertical line represents an intron, a and b represent two ABCE genes in Arabidopsis thaliana. The blue panel represents the intron position conserved between Tetrahymena and Arabidopsis ABCE genes. [file 1471-2148-10-330-S4.TIFF]

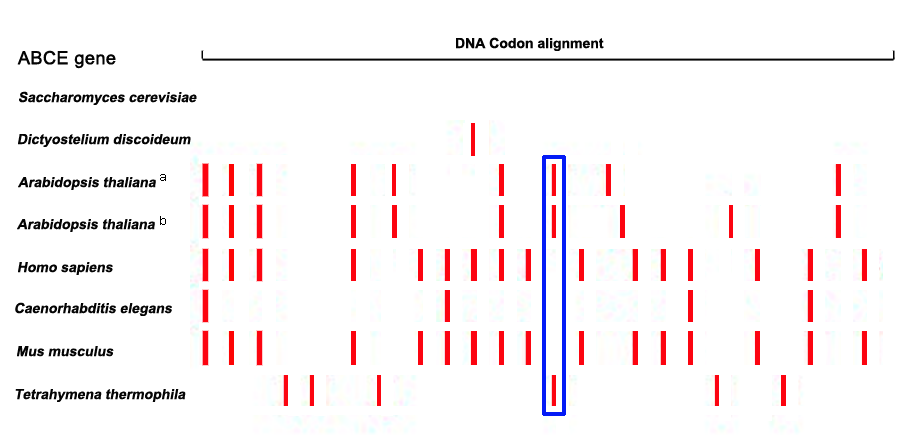

Supplement: Additional file 5 — Expression profiles of ABCD1 and ABCD2. 20 time points of the three physiological/development stages of Tetrahymena. For growing cells, L-l, L-m and L-h correspond to ~1×10^5^ cells/ml, ~3.5×10^5^ cells/ml and ~1×10^6^ cells/ml, respectively. For starvation, ~2×10^5^ cells/ml were collected at intervals of 0, 3, 6, 9, 12, 15 and 24 hours (referred to as S-0, S-3, S-6, S-9, S-12, S-15 and S-24, respectively). For conjugation, equal volumes of B2086 and CU428 cells were mixed in culture, and samples were collected at intervals of 0, 2, 4, 6, 8, 10, 12, 14, 16 and 18 hours after mixing (referred to as C-0, C-2, C-4, C-6, C-8, C-10, C-12, C-14, C-16 and C-18, respectively). [file 1471-2148-10-330-S5.TIFF]
