# Supplementary material for: Genome-wide identification and evolution of ATP-binding cassette transporters in the ciliate Tetrahymena thermophila: A case of functional divergence in a multigene family
Source: BMC Evol Biol. 2010 Oct 27;10:330. doi: 10.1186/1471-2148-10-330 (PMC2984421; doi:10.1186/1471-2148-10-330)
Supplement: Additional file 6 — Different candidates for co-expression of genes between two genes in each pair. The quotient (high number/low number) and the difference (high number/low number) of candidate co-expressed gene number between two genes in each pair have been calculated. a: the percentage of pairs in which the quotient > 2. b: the percentage of pairs in which the difference > 100. [file 1471-2148-10-330-S6.DOC]

| **Data Set** | **Pairs** | **Quotient^a^ >2** | **Difference^b^ >100** |
| --- | --- | --- | --- |
| ABCA SET1 | 120 | 78.3% | 75.0% |
| ABCA SET2 | 45 | 80.4% | 37.0% |
| ABCB SET1 | 28 | 46.4% | 39.3% |
| ABCB SET2 | 28 | 82.1% | 75.0% |
| ABCC SET1 | 105 | 83.8% | 61.9% |
| ABCC SET4 | 325 | 80.0% | 54.9% |
| ABCG SET1 | 496 | 79.9% | 71.4% |
